# Garlic, from Remedy to Stimulant: Evaluation of Antifungal Potential Reveals Diversity in Phytoalexin Allicin Content among Garlic Cultivars; Allicin Containing Aqueous Garlic Extracts Trigger Antioxidants in Cucumber

**DOI:** 10.3389/fpls.2016.01235

**Published:** 2016-08-25

**Authors:** Sikandar Hayat, Zhihui Cheng, Husain Ahmad, Muhammad Ali, Xuejin Chen, Mengyi Wang

**Affiliations:** ^1^Department of Vegetable Science, College of Horticulture, Northwest A&F University, YanglingChina; ^2^School of Horticulture Landscape Architecture, Henan Institute of Science and Technology, XinxiangChina

**Keywords:** Allicin, genetic diversity, antifungal, biostimulant, antioxidant enzymes, HPLC

## Abstract

Garlic has the charisma of a potent remedy and holds its repute of a therapeutic panacea since the dawn of civilization. An integrated approach was adopted to evaluate the genetic diversity among Chinese garlic cultivars for their antifungal potency as well as allicin content distribution and, furthermore; a bioassay was performed to study the bio-stimulation mechanism of aqueous garlic extracts (AGE) in the growth and physiology of cucumber (*Cucumis sativus*). Initially, 28 garlic cultivars were evaluated against four kinds of phytopathogenic fungi; *Fusarium oxysporum, Botrytis cinerea, Verticillium dahliae* and *Phytophthora capsici*, respectively. A capricious antifungal potential among the selected garlic cultivars was observed. HPLC fingerprinting and quantification confirmed diversity in allicin abundance among the selected cultivars. Cultivar G025, G064, and G074 had the highest allicin content of 3.98, 3.7, and 3.66 mg g^-1^, respectively, whereas G110 was found to have lowest allicin content of 0.66 mg g^-1^. Cluster analysis revealed three groups on the basis of antifungal activity and allicin content among the garlic cultivars. Cultivar G025, G2011-4, and G110 were further evaluated to authenticate the findings through different solvents and shelf life duration and G025 had the strongest antifungal activity in all conditions. minimum inhibitory concentration and minimum fungicidal concentration of Allicin aqueous standard (AAS) and AGE showed significant role of allicin as primary antifungal substance of AGE. Leaf disk bioassay against *P. capsici* and *V. dahliae* to comparatively study direct action of AGE and AAS during infection process employing eggplant and pepper leaves showed a significant reduction in infection percentage. To study the bioactivity of AGE, a bioassay was performed using cucumber seedlings and results revealed that AGE is biologically active inside cucumber seedlings and alters the defense mechanism of the plant probably activating reactive oxygen species at mild concentrations. However, at higher concentrations, it might cause lipid peroxidation and membrane damage which temper the growth of cucumber seedlings. At the outcome of the study, an argument is advanced that current research findings provide bases for cultivar selection in antifungal effectivity as well as genetic variability of the cultivars. Allicin containing AGE can be used in specialized horticultural situations such as plastic tunnel and organic farming as a bio-stimulant to enhance cucumber growth and attenuate fungal degradation of agricultural produce.

## Introduction

Plants and plant-derived produce are among the prime utilities of mankind for food, shelter and cure since the dawn of civilization and it wouldn’t be inappropriate to state that the use of medicinal plants predates written human history (Harrison et al., 2015). Among these medicinal plants, garlic (*Allium sativum* L.) has secured its repute of a therapeutic panacea (Ankri and Mirelman, 1999). Garlic has been documented to possess antimicrobial (Feldberg et al., 1988; Ankri and Mirelman, 1999; Coppi et al., 2006; Ayazi, 2011; Wallock-Richards et al., 2014), anticancer (Thomson and Ali, 2003; Rana et al., 2011; Lee et al., 2013), antidiabetic (Lee et al., 2009), antiviral potential (Corzo-Martínez et al., 2007) and nevertheless, has ability to cope with cardiac complications (Lawson et al., 1992; Borek, 2001). Various organosulfur compounds such as DADS, DATS, DAS, Ajoene etc., have been suggested to contribute to the biological properties of garlic (Martins et al., 2016). However, the activity of a compound may vary depending on the methods and conditions employed for extraction (Ankri and Mirelman, 1999; Fujisawa et al., 2008; Mohammed et al., 2013; Martins et al., 2016). Therefore, to understand the biological activity of a compound, appropriate processing techniques and conditions that account the bioavailability are vital to be considered and explored (Rahman and Lowe, 2006). The putative antimicrobial constituent of garlic (Diallyl Thiosulfinate) was identified by Cavallito in 1944 and was given the name allicin. During the last few decades, efforts have been put together to identify (Fujisawa et al., 2008), isolate (Farías-campomanes et al., 2014) and utilize allicin from garlic (Banerjee et al., 2003; Bhuiyan et al., 2015). Although researchers have paid considerable attention to isolate and identify bioactive compounds of garlic which account for its marvelous therapeutic repute, less reports are available regarding the diversity for abundance of the active allelochemicals within different garlic ecotypes and very few literature document the considerable differences accounted for total phenolic compounds (Chen et al., 2013). Jo et al. (2012) used SSR markers to explore genetic diversity among selected garlic cultivars and reported that the diversity among the garlic cultivars was correlated to the geographic distribution. Due to cultivation pattern, garlic is usually sown in single clove in nearly all types of environments round the globe which offers a great chance of genotypic plasticity in garlic (Horníčková et al., 2010). However, to date, reports that document the genetic diversity in garlic cultivars of Chinese origin are few and far between.

In cropping system, garlic has been reported to have spectacular effects on growth of the receiver plant (Lin et al., 1964; Zhi-hui, 2011; Han et al., 2013) and also, its ability to help overcome continuous cropping obstacles in eggplant (Wang et al., 2015), cucumber (Xiao et al., 2012, 2013) and pepper (Ahmad et al., 2013) has been well established. However, utilization of garlic derived botanicals which bear antifungal potential, need to be explored particularly in specialized horticultural practicing situation such as plastic tunnel farming systems where production is sometimes significantly limited due to microbial problems in general and fungal infections in particular. Therefore, in order to formulate preparations for biocontrol agents, identification of bioactive compounds, their extraction, and furthermore, clarifying their bioactivity needs careful evaluation (Mukerji, 2006).

During the course of agricultural expansion, the infirmities and hurdles of biotic origin led to the utilization of fungicides, bactericides and pesticides etc. (Wilson and Tisdell, 2001; Aktar et al., 2009) for sustainable agricultural production. The frequent and overuse of these chemicals, however, brought hazardous consequences to the farming community (Bolognesi, 2003; Gilden et al., 2010; Mostafalou and Abdollahi, 2013; Sugeng et al., 2013). Nonetheless, some microbes evolved resistance to the commercial antimicrobials (Staub, 1991; Steffens et al., 1996; Ma and Michailides, 2005; Deising et al., 2008) asserting room for botanicals of organic nature that are more promising, cost effective, less hazardous, and beneficial to the farming as well as consumers community (Gurjar et al., 2012; Yoon et al., 2013). The use of garlic bulb extracts has been reported to enhance protection against a variety of diseases (Haggag, 2007; Ting-ting et al., 2011) as well as some reports also mention the bio-stimulatory effects of garlic (Muhammad and Akladious, 2014; Al-obady, 2015). However, very few reports advocate the ability of garlic bulb extracts as biological activator for induced state of defense in the plants.

Recently, scientists are evaluating microbial degradation of waste products particularly those of the plastic and pesticide residual degradation through mycological approaches and are therefore suggesting the application of fungal colonization for degradation of these hazardous chemicals in order to ensure safe environments and ecosystems (Das and Chandran, 2011; Amirjani, 2012). However, due to the importance of crop production for sustainable agriculture, it is inevitable to control phytopathogenic microorganisms. Acknowledging the necessity of both the situations mentioned above, identification, preparation and utilization of organic fungicides or biostimulants would be of great potential that could ensure crop quality as well as promise less hazards to the environment with minimum or no residual effects particularly in specialized production units where these residual products pose significant threats both to the farmers as well as plant communities.

Piecing together the broad spectrum antimicrobial potential, strong allelopathic capacity and ease of availability, garlic offers a very attractive option to consider as bio-stimulator or inducer for enhanced production and significant protection against variety of fungal disorders. Exploring and identifying the actual bioactivity, however, require elaborated study on these particular allelochemicals of garlic. Current research work is thus an effort put forward to evaluate the Phytoalexin allicin distribution among different garlic ecotypes in order to explore possible genetic diversity and to advocate allicin containing garlic bulb extracts as plant activator for enhanced production as well as test its capability as a potent biofungicide. We have employed multidisciplinary approaches using analytical chemistry, microbiology and bio-physiology for investigations and our findings demonstrate that from remedy to bio-stimulant, aqueous garlic extracts (AGE) offer a great deal both as biofungicide as well as bio-stimulator for better growth in cucumber, particularly those grown under plastic tunnel or greenhouse conditions.

## Materials and Methods

### Garlic Cultivars, Aqueous Extract Preparation and Allicin Quantification

Fresh, uniform sized bulbs of 28 garlic cultivars were selected from the garlic germplasm NWSUAF Yangling, Shaanxi, China. The selected cultivars were stored at -20°C until further use. Aqueous extracts were made according to Ting-ting et al. (2011) with slight modifications. Briefly, randomly selected 10 g of sample from each cultivar was ground in a sterile mortar and pistil and then homogenized in 100 mL distilled water. The homogenate was further centrifuged at 10,000 rpm and the supernatant was collected and filtered through 0.22 μm pore filter. Serial dilutions were further carried out accordingly for each bioassay as required. We prepared fresh extract for each time so as to assure the maximum output.

Allicin aqueous standard (ASB-0001535-005) was purchased from ChromaDex International USA. For HPLC, we followed standard procedure (Wallock-Richards et al., 2014) with some modifications. Serial dilutions of Allicin aqueous standard (AAS) from 3900–39 μg mL^-1^ were prepared for calibration of RP-HPLC method (Column dimensions: 150 mm × 4.6 mm, C18 Diamonsil, Dikma technologies). The elution conditions were as: isocratic elution with MeOH/water (60:40, v/v) at 1 mL min^-1^ with UV detection at 240 nm, 25°C with injection volume of 10 μL for all samples. Mass of allicin was confirmed in the AAS by direct infusion mass spectrometry.

### Fungal Strains

Four fungal species were employed in this research; *Botrytis cinerea, Fusarium oxysporum, Phytophthora capsici*, and *Verticillium dahliae.* These fungal strains were maintained at Potato Dextrose Agar medium incubated at 28°C. For bioassay, 7–10 days old cultures were used.

### Determination of Minimum Inhibitory Concentration (MIC) and Minimum Fungicidal concentration (MFC)

To investigate the minimum inhibitory concentration (MIC) and minimum fungicidal concentration (MFC) of AGE and AAS, we followed standard protocols (Liu et al., 2002; Tao et al., 2014) with slight modifications to confirm the role of allicin as a prime antifungal substance in the garlic extracts employed in current research. AGE was prepared as discussed above and further dilutions were carried out ranging from 0.001 to 10% (where 100% represents 10 g of garlic homogenized in 10 mL of distilled water), whereas AAS was diluted ranging from 0.0039 to 390 μg mL^-1^. Potato Dextrose Agar medium and Potato Dextrose Broth were used to perform the experiments. Macrodilution technique according to Tao et al. (2014), whereas microdilution technique was practiced according to the methods stated by Liu et al. (2002). The concentration that inhibited the fungal growth by 50% was regarded as MIC50 whereas the concentration which inhibited the fungal growth for 90% was regarded as MIC90. The minimum concentration which completely inhibited fungal growth after re-culturing onto a fresh medium after the stated incubation period, was confirmed as MFC or MFC.

### Leaf Disk Bioassay

The methods of Liu R. et al. (2014) and Wan et al. (2015) were followed to perform a leaf disk bioassay in order to test the allicin-containing AGE and pure AAS inhibitive effect on *V. dahliae* and *P. capsici*. Fresh, fully expanded leaves were selected from eggplant and pepper and leaf disks were prepared using a sterilized 9 mm Cork borer. These disks were surface sterilized with bleach (0.01%) and washed three times with doubled distilled water. AGE with three different concentrations (100, 50, and 25 mg mL^-1^) were prepared and their respective allicin content was quantified (3.9 mg g^-1^, 0.39, and 0.039 mg g^-1^, respectively) according to HPLC quantification. AAS was purchased from Chromadex International USA and similar concentrations were prepared. Leaf disks were immersed in the respective treatment (10 mL contained in a test tube) for 2 min and then placed on a wet filter paper in a glass petri dish. For control, distilled water was used. 20 μL of conidial suspension adjusted to 5 × 10^-4^ were applied to each disk and the petri dishes were covered with lids with covering plastic film in order to maintain humidity and incubated as described by Wan et al. (2015). After 5 days, data were recorded for disease incidence, disease severity % and severity index %. Disease incidences were quantified by determining the number of disks with sporulation per total number of disks. To quantify disease severity %, percentage of leaf disk area with lesions was measured. Severity index % was quantified as described by Liu R. et al. (2014). Pictures were digitally analyzed using ImageJ software (Abràmofff et al., 2005).

### Cucumber Bioassay

A bioassay was performed in a glasshouse pot experiment trial to assess the biological triggering mechanism of AGE in the growth and physiology of cucumber plants. Cucumber seeds were grown in plastic trays in a growth chamber until germination. Upon second true leaf stage, these seedlings were transferred to pots and maintained in glasshouse facility. AGE was prepared as earlier described and diluted to 50, 150, and 300 μg mL^-1^, respectively. One week after transplanting, these extracts were sprayed on the cucumber seedlings (20 mL plant^-1^), while spraying distilled water as control treatment. A randomized complete block design was used to perform the experiment with three replications. Each treatment consisted of 10 seedlings. After 20 days, data were recorded for plant height; root length, stem diameter, and samples were taken and immediately stored in ice box for physiological assessment of superoxide dismutase (SOD), peroxidase (POD), catalase (CAT), and malondialdehyde (MDA) content.

### Determination of Antioxidants (SOD, POD, and CAT) and MDA Content

We followed a standard procedure stated by Wang et al. (2015) to perform the antioxidant enzymes and MDA analysis. Briefly, leaf samples (0.500 g) were ground with 2 mL of cold extraction buffer (0.05 M phosphate buffer, pH 7.8), and the entire mixture was transferred to centrifuge tubes with another 6 mL of the same extraction buffer and centrifuged for 20 min at 10,000 × *g*. The supernatant was used to determine the content of MDA and enzyme activities for each treatment; the measurements were performed in triplicate.

The MDA content was measured using the thiobarbituric acid (TBA) reaction. Two milliliter of the extract supernatant was mixed with 2 mL 0.6% (w/v) TBA solution dissolved in 5% (v/v) trichloroacetic acid (TCA), heated in boiling water for 10 min, and then cooled to allow the flocculate to sediment. The supernatant was used for the spectrophotometric determination of MDA. The absorbance at the wavelength of 450 and 532 nm was measured and subtracted from the absorbance at 600 nm. MDA content was expressed as the amount of substance per gram of fresh leaves (nmol⋅g^-1^Fw). Total SOD activity was estimated by the inhibition of the photochemical reduction of nitro blue tetrazolium (NBT). The reaction mixture contained 1.5 mL 0.05 M phosphate buffer (pH 7.8), 0.3 mL 0.1 mmol⋅L^-1^ EDTA-Na_2_, 0.3 mL 0.13 mol⋅L^-1^ methionine, 0.3 mL 0.75 mmol⋅L^-1^ NBT, 0.3 mL 0.02 mmol⋅L^-1^ riboflavin, 0.05 mL enzymatic extract, and 0.25 mL distilled water in a total volume of 3 mL for the reaction mixture. After exposure to fluorescent light (86.86 μmol⋅m^-2^⋅s^-1^) for 10–20 min (end point determined by the color of the reaction solution), the absorbance was recorded at the wavelength of 560 nm. SOD activity was determined as 50% inhibition of the NBT reduction caused by the superoxides generated from the reaction of photo-reduced riboflavin and oxygen. The total SOD activity was expressed in units per gram of fresh leaves (U⋅g^-1^ FW). The guaiacol method was used for the determination of POD activity. A reaction mixture was prepared using 50 mL 0.05 M phosphate buffer (pH 7.8), 28 μL guaiacol, and 19 μL 30% H_2_O_2_ (v/v); 3.5 mL of the reaction mixture solution was placed into a cuvette with a 1 cm path length. The increase in absorbance at the wavelength of 470 nm was recorded over 3 min at 30 s intervals after the addition of 0.5 mL enzyme extract. The results were presented as D470 per minute per gram of fresh leaves (U⋅g^-1^⋅min^-1^). The CAT reaction mixture comprised 0.1 ml enzyme extract, 1.9 ml of 200 mM phosphate buffer (pH 7.0), and 1 ml 0.3% H_2_O_2_, and enzyme activity was assayed by measuring the reduction of H_2_O_2_ at 240 nm for 3 min with a spectrophotometer. The activity of CAT is presented as OD 240 nm min^-1^ g^-1^.

### Statistical Analysis

Each experimental data was analyzed statistically using analysis of variance ANOVA. Comparison among means were performed according to Least significant difference LSD with 0.05 level of significance.

## Results

### Antifungal Potential of AGE and Diversity in the Abundance of Phytoalexin Allicin Content among Different Garlic Cultivars

**FIGURE 1:**
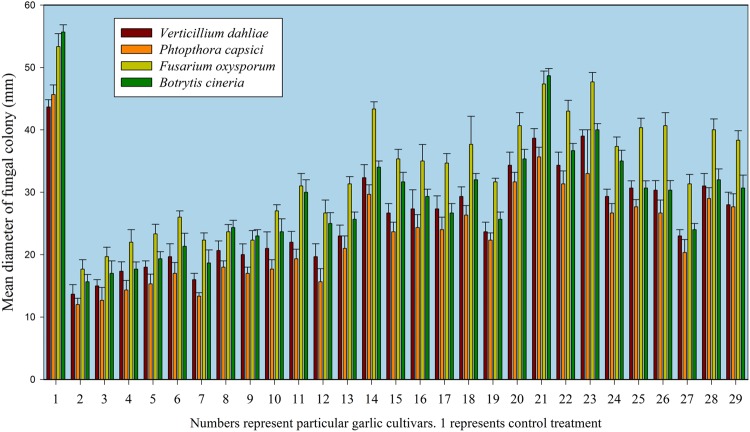
**Antifungal activity of selected garlic cultivars against phytopathogenic fungi.** Data represent means and standard errors of triplicates for fungal colonial diameter in mm while numbers represent the respective garlic cultivars. 1 represents control treatment.

**FIGURE 2:**
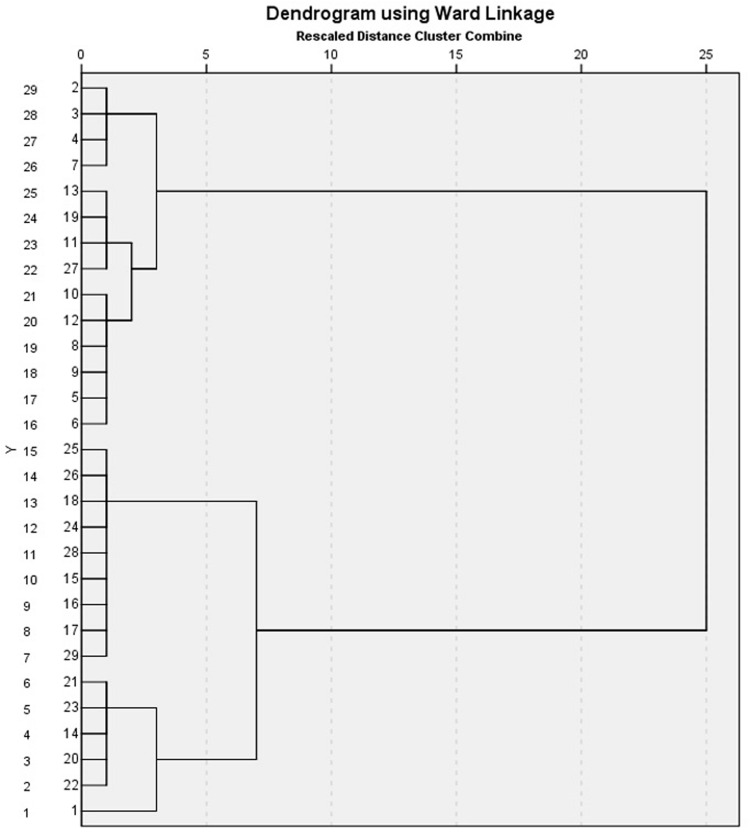
**Dendogram of clusters for the respective garlic cultivars.** Numerical number represent particular cultivar. 2, 3, 4, 5, 6, 7, 8, 9, 10, 11, 12, 13, 19, and 27 (G025, G064, G74-X, G002, G022, G008, G107, G2012-3, G2012-1, G2010-1, G052, G103, G057, and G039). Strong, 15, 16, 17, 18, 24, 25, 26, 28, and 29 (G2011-5, G2011-12, G2010-5, G2011-4, G090, G105, G2010-6, G026, and G057). Moderate, while 14, 20, 21, 22, and 23 (G085, G083, G005, G110, and G059) are clustered as Weak potential cultivars.

**FIGURE 3:**
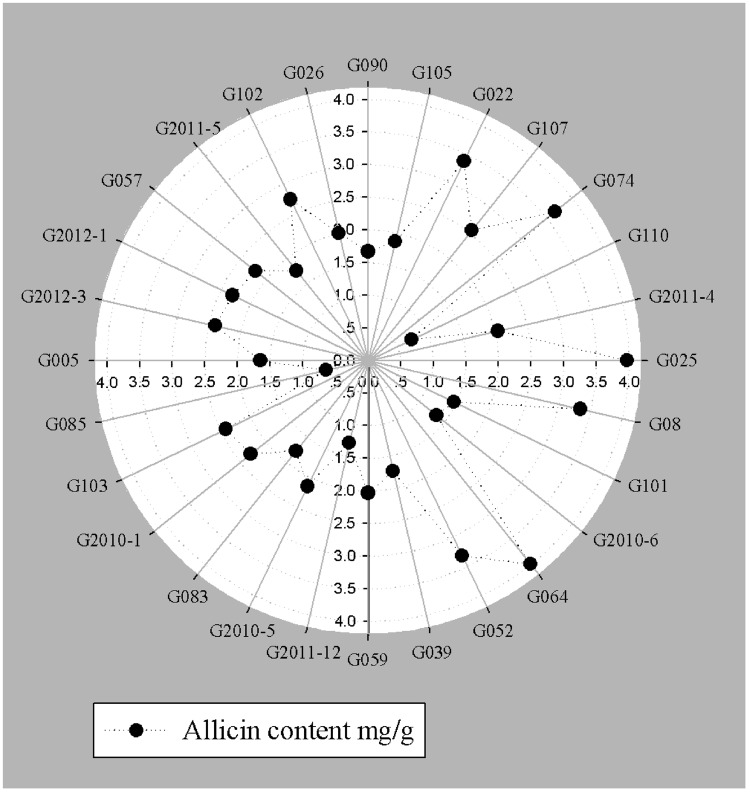
**Allicin content (mg g^-1^) fresh weight of garlic bulb quantified by HPLC**.

**FIGURE 4:**
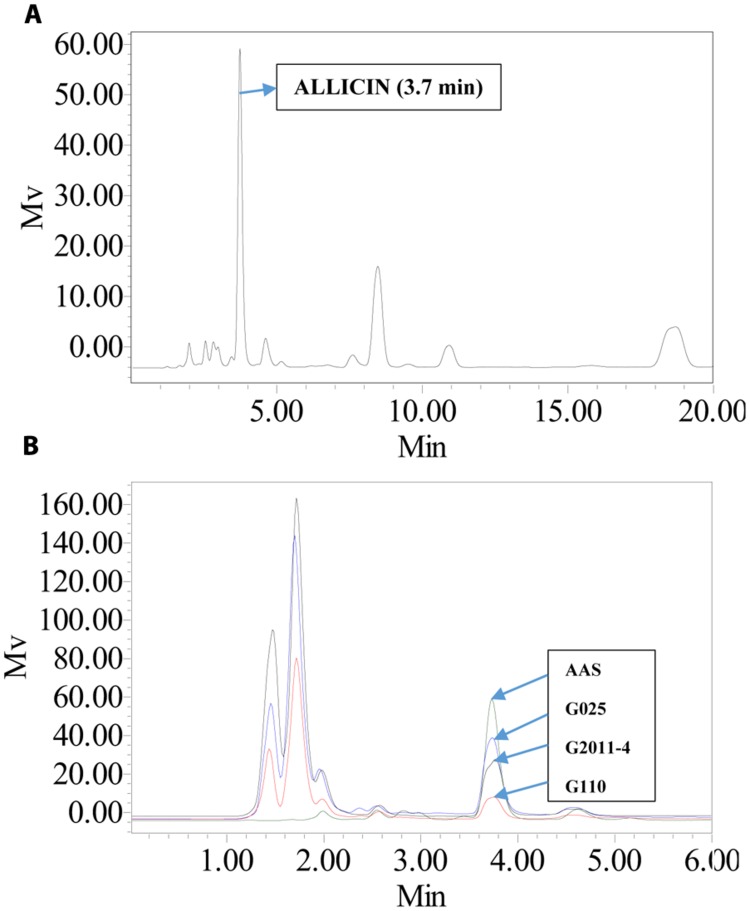
**HPLC chromatograms of allicin aqueous standard (AAS) and aqueous garlic extracts (AGE). (A)** AAS was analyzed using a C18 column with UV detection at 240 nm. The standard eluted at 3.7 min. AAS had an observed m/z of 163. **(B)** Comparison of AAS and AGE for G025, G2011-4, and G110 under same elution conditions. Height of peak depicts the difference in allicin abundance.

**FIGURE 5:**
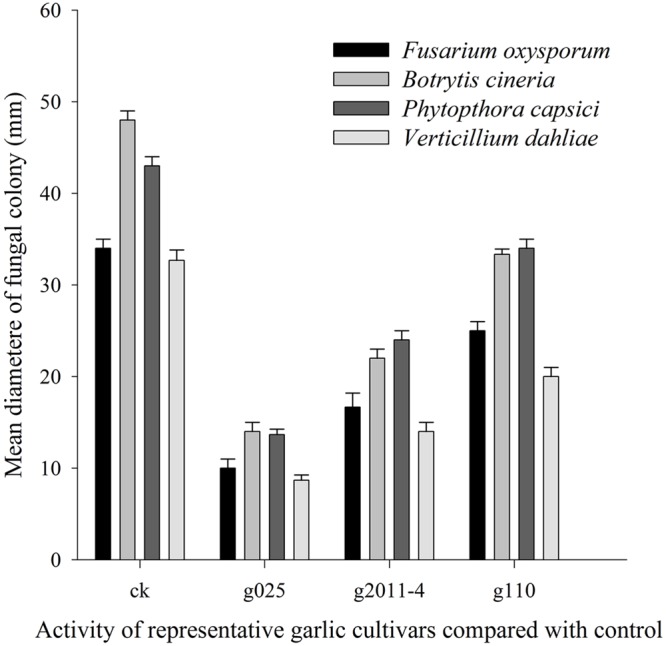
**Antifungal activity of AGE for representative garlic cultivars against selected fungal strains.** The means and standard errors are represented in bars. All the data were significantly different at *P* = 0.05 using LSD.

**FIGURE 6:**
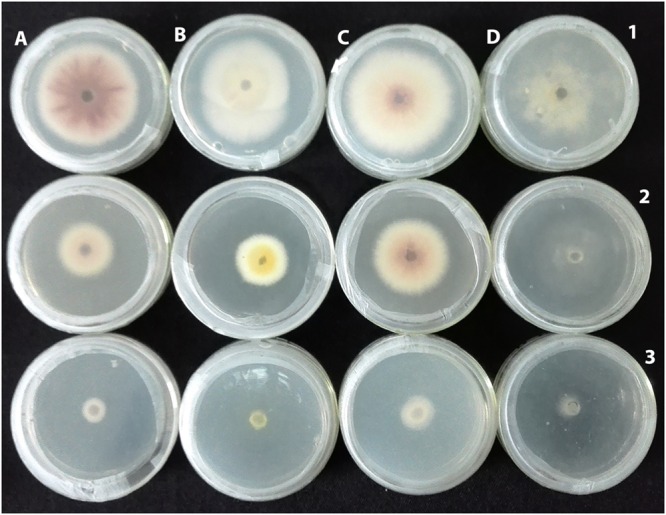
**Antifungal activity of G025 linear to the concentrations of its extracts used. (A)** *Fusarium oxysporum*, **(B)** *Verticillium dahliae*, **(C)** *Phytopthora capsici*, and **(D)** *Botrytis cinerea* at 25 mg mL^-1^ **(1)**, 50 mg mL^-1^ **(2)** and 100 mg mL^-1^ **(3)**.

An integrated approach was adopted to classify garlic cultivars based on antifungal potential, quantify the allicin content and advance the preparations for a potent and handy biofungicide against broad spectrum fungi. We primarily screened 28 garlic cultivars to test the genetic variability between various cultivars on the basis of antifungal activity. Capricious antifungal potency was observed among these garlic cultivars (**Figure 1**). The strongest antifungal activity was recorded for G025 reducing the fungal colonial growth to 12.33 mm, followed by G064 (12.66 mm) against fungal strain *F. oxysporum*, while the largest diameter next to the control treatment was 47.66 mm for G083 exhibiting the lowest antifungal activity against *P. capsici*. Although the fungal strains individual values differed *per se*; however, the overall results showed a similar trend in the data. Garlic cultivars G025, G064, G022, and G074 had the strong antifungal potential followed by G102, G008, G107, G2012-3, G2011-4, G2012-1, G2010-1, G052, G103, G101, G2011-12, G2010-5, G2011-5, G039, G026, G2010-6, G110, G085, G057, G005, G090 G059, G083, and G105, respectively. The data depicted cliques of colonial growth suggesting a difference between the antifungal activities of the selected garlic cultivars. More or less the same pattern was observed for each fungal strain pertaining different cultivars which suggests the broad spectrum activity of AGE. Cluster analysis revealed three distinct groups classified as strong, medium and low based on the antifungal potential (**Figure 2**). To evaluate the allicin content in the selected garlic cultivars and to justify the results, HPLC was performed. Garlic cultivar G025 and G064 had the highest allicin content of 3.98 and 3.97 mg g^-1^, respectively, while the lowest allicin content was observed in G085 and G110 which was 0.74 and 0.66 mg g^-1^ of the garlic bulb, respectively (**Figure 3**). Allicin in the AAS eluted at 3.7 min (**Figure 4A**) and was confirmed by mass spectrophotometry with m/z = 162.9. The peak eluted at 3.7 min in the AGE was thus confirmed to be allicin. Interestingly, a cadence of the allicin content in different cultivars was observed (**Figure 4B**). Moreover, the antifungal activity of the studied garlic cultivars was linear in correlation to the abundance of the respective allicin content. We further selected one representative cultivar from each cluster and performed different *in vitro* bioassays to authenticate our findings and study for solvent choice for extraction and shelf life durations on the activity. **Figure 5** depicts the efficacy of AGE for the representative cultivars from each group against the selected fungal strains. G025 gave the highest antifungal activities among these selected cultivars. The efficacy of the extracts declined after 24 h during storage evaluation, while distilled water and ethanolic extracts gave considerably higher activity as compared to di-ethyl ether in the solvent evaluation study. The concentrations of ethanol did not affect the efficacy of the AGE (Data not shown). **Figure 6** represents the antifungal activity of G025 against the selected fungal strains and it could be observed that the antifungal potential correlates with the concentration employed. Moreover, **Table 1** represents the MIC and MFC of AGE and pure allicin AAS against the selected fungal strains. Both the macrodilution and microdilution method revealed significant role of allicin as the prime antifungal substance in the AGE and the MICs range from 1 to 5% depending on the fungal strains and experimental procedures. The complete inhibition or fungicidal concentration was 10% in both the procedures against mycelial plugs as well as spore suspensions of the fungi. Pure allicin AAS showed an MIC of 39–195 μg mL^-1^ allicin content which coincides with the amount of allicin observed in our AGE through HPLC analysis. Nevertheless, the MFC for AAS was observed to be 390 μg mL^-1^ and thus our findings about the antifungal potential of AGE are therefore strongly suggested to be because of the allicin content of these cultivars.

**Table 1:** Minimum inhibitory concentration (MIC) and minimum fungicidal concentration (MFC) of AGE and AAS tested against the selected fungal strains.

	Microdilution		Macrodilution		
	MIC50	MIC90	MIC50	MIC90	MFC
AGE	1%	10%	5%	10%	10%
AAS	39 μg mL^-1^	390 μg mL^-1^	195 μg mL^-1^	390 μg mL^-1^	390 μg mL^-1^

*Aqueous garlic extracts was diluted into various concentrations where 100% means 10 g of garlic per 10 mL of distilled water. AAS was diluted from the initial 3.9 mg mL^-*1*^ into various dilutions. MIC 50 is the concentration which inhibits fungal growth by 50% whereas MIC90 is the concentration which inhibits fungal growth by 90%. MFC represents minimum fungicidal concentration.*

### Leaf Disk Bioassay against *Verticillium dahliae* and *Phytopthora Capsici*

**FIGURE 7:**
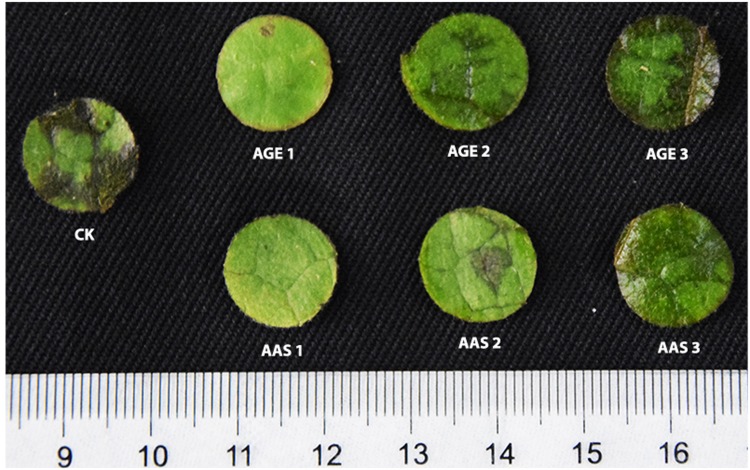
**Leaf disk showing antifungal activity of pure allicin AAS and AGE.** CK represents Control, AGE1 (100 mg mL^-1^), AGE2 (50 mg mL^-1^), AGE3 (25 mg mL^-1^), AAS1 (3.9 mg g^-1^), AAS2 (0.39 mg g^-1^), and AAS3 (0.039 mg g^-1^). The amount of AAS is relative to that of allicin content of AGE quantified for G025 through HPLC.

To test the preventive activity of allicin-containing AGE against fungal infection on plant surface, leaf disk bioassay was performed. Statistical analysis of results confirmed that both the AAS as well as the AGE attenuated the pathogenicity of *P. capsici* and *V. dahliae* and hampered the infection process on the leaf disks (**Table 2**). The concentration of allicin either as AAS or, as a constituent of AGE, limited the fungal infection resulting in considerably lower disease severity as well as severity indexes suggesting that allicin might be the primary (if not the only), active antifungal substance that actively reduce the fungal infection on plant surface. Our findings revealed that AGE at 100 mg mL^-1^ had the lowest disease severity as compared to the control treatment. However, at lower concentrations, the prevention effect decreased (**Figure 7**).

**Table 2:** Leaf disk bioassay for comparative evaluation of aqueous garlic extracts (AGE) and Allicin (AAS) against *Verticillium dahliae* and *Phytopthora capsici*.

Treatment	EGG PLANT		PEPPER	
	Disease severity %	Severity index %	Disease severity %	Severity index %
CK	85.00 ± 1.15^a^	45.34 ± 0.67^a^	84.67 ± 1.76^a^	46.18 ± 1.08^a^
AGE 1	14.33 ± 1.33^d^	12.48 ± 0.58^d^	12.67 ± 0.88^d^	12.63 ± 1.52^d^
AGE 2	43.33 ± 1.67^c^	26.83 ± 1.04^c^	38.67 ± 1.2^c^	26.27 ± 1.17^c^
AGE 3	73.33 ± 2.03^b^	35.25 ± 0.81^b^	68.00 ± 1.53^b^	40.74 ± 1.41^b^
AAS 1	13.66 ± 0.88^d^	11.60 ± 1.08^d^	10.67 ± 0.67^d^	13.7 ± 1.54^d^
AAS 2	39.66 ± 2.40^c^	26.85 ± 1.76^c^	37.67 ± 1.45^c^	27.53 ± 1.31^c^
AAS 3	77.00 ± 1.15^ab^	35.60 ± 1.24^b^	73.00 ± 1.15^b^	36.03 ± 1.96^b^

*CK represents Control, AGE1 (100 mg mL^-*1*^), AGE2 (50 mg mL^-*1*^), AGE3 (25 mg mL^-*1*^), AAS1 (3.9 mg g^-*1*^), AAS2 (0.39 mg g^-*1*^), and AAS3 (0.039 mg g^-*1*^). The amount of AAS is relative to that of allicin content of AGE quantified for G025 through HPLC. The means and SE are represented for comparison. Letters represents significant difference at *p* = 0.05.*

### Bioassay on Cucumber Seedlings in Glasshouse Experiment

**FIGURE 8:**
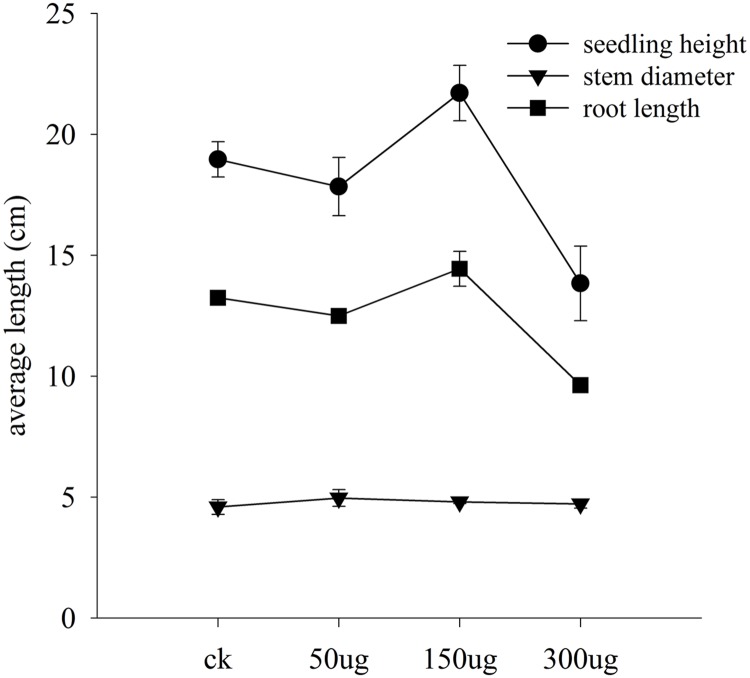
**Bioactivity of AGE in cucumber seedlings.** Data represent means and standard errors for triplicates of selected morphological characters of cucumber seedlings. Seedling height and root length data show difference at 0.05 level of significance.

**FIGURE 9:**
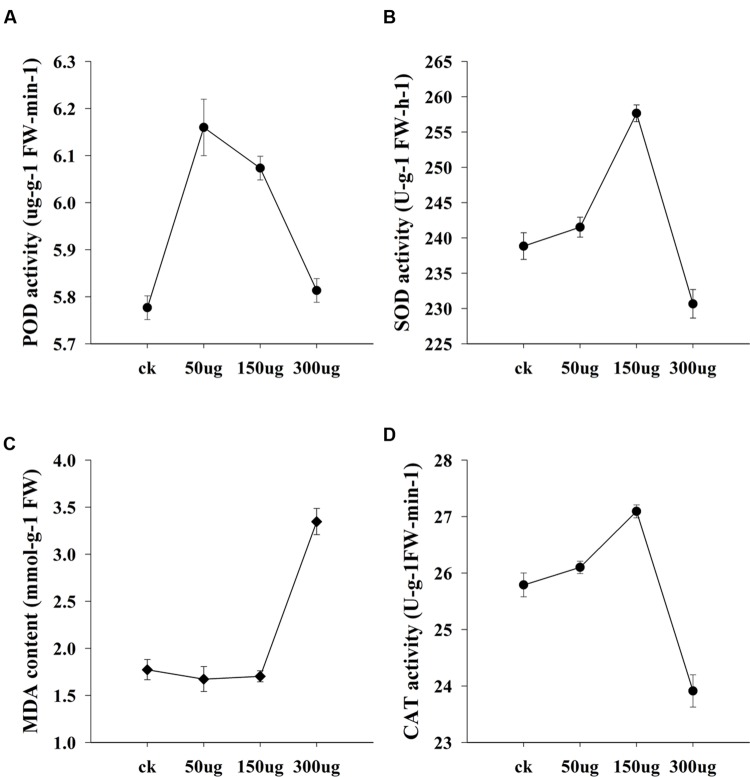
**Bioactivity of AGE in the physiology of cucumber seedlings.** The means and standard errors of biological replicates are shown. Significance in variance was analyzed at 0.05 level of significance. **(A)** POD activity of cucumber seedlings, **(B)** SOD activity of cucumber seedlings, **(C)** MDA content of cucumber seedlings and **(D)** CAT activity of cucumber seedlings.

To test the possibility that allicin-containing AGE biologically trigger the plant physiology, bioassay was performed in a glasshouse on cucumber seedlings. Data for morphological traits is interpreted in **Figure 8**. The test plants exhibited a dose dependent response to the applied AGE concentrations. Plant height and root length altered with the variable concentrations of AGE applied while plant diameter in current research findings gave no significant results. Data for plant height exhibited an increase and decrease trend with toward variable concentrations. AGE concentration at 50 μg mL^-1^ did not show any significant response and maximum seedling height was observed at concentration of 150 μg mL^-1^ AGE but when the concentration was doubled to 300 μg mL^-1^, the seedling height drastically decreased. Similar response was observed in root length, i.e., increased at 150 μg mL^-1^ AGE but at 300 μg mL^-1^, root length was reduced as compared to the control treatment. The physiological data for cucumber seedlings is interpreted in **Figure 9**. Statistical analysis revealed that AGE affected the enzymes and MDA content of cucumber seedlings at variable concentrations. POD activity showed variations to different concentration levels of AGE (**Figure 9A**) and maximum activity was observed in 50 μg mL^-1^ followed by at 150 μg mL^-1^ but at the highest concentration of AGE, i.e., 300 μg mL^-1^, the POD activity decreased as compared to control treatment. The data for SOD is presented in **Figure 9B** and the maximum activity was observed at 150 μg mL^-1^ concentration of AGE as compared to control. However, when the concentration of AGE was 300 μg mL^-1^, the SOD activity drastically decreased. Malondialdehyde (MDA) content is depicted in **Figure 9C**. At low concentrations, we observed no significant alterations in the MDA content between the control and treated seedlings but at 300 μg mL^-1^ concentration of AGE, abundance in the MDA content was observed suggesting the onset of a stress conditions in the seedlings. CAT activity also gave significant response when the seedlings encountered variable dosage of AGE (**Figure 9D**), and maximum activity was observed at 150 μg mL^-1^, while the lowest significant value for CAT activity was observed at 300 μg mL^-1^.

## Discussion

The antifungal potency of garlic derived allicin might be no novel phenomenon, but the capricious antifungal activity and diversity in the abundance of allicin content within the garlic cultivars are vital findings of current research work that allow us to postulate the genetic diversity within these cultivars. AGE significantly inhibited the growth of fungi in all the bioassays performed. Allicin (Diallyl-thiosulfinate), the prime antimicrobial, is the principal among various organosulfur compounds that constitute garlic bulb extracts (Bayan et al., 2014). Although it is arbitrary to attribute allicin as the sole antifungal component in the current study, yet there is possibility of its major contribution due to the fact that allicin is the primary and predominant bioactive compound present in freshly prepared aqueous extracts of garlic whereas various other sulfur-containing compounds such as DADS or DAS, either are produced during storage of AGE or dominate mostly the oils extracts of garlic (Kyung, 2012). It was observed that the antifungal activity differed between various cultivars and the effect was linear to the abundance of allicin content therein, and moreover, the MIC and MFC ranges of pure allicin AAS coincided with the content of allicin quantified in the AGE through HPLC analysis. Therefore, it can be suggested that allicin is the major or principal antifungal component of freshly prepared AGE. The efficacy of the AGE, however, faded with prolonged shelf storage (data not shown). Previous findings support our ideas who reported that the primary antifungal substance “allicin,” is highly unstable and readily decompose into various organosulfur compounds such as DAS, DADS, DATS etc., (Rahman and Lowe, 2006; Yun et al., 2014) and prolonged storage conditions particularly effect the stability of allicin (Fujisawa et al., 2008; Martins et al., 2016). Therefore, the decline in the efficacy of the AGE might be due to decomposition of the allicin content into some other organosulfur compounds having low antifungal potential. The possible mechanics involved in delimiting the fungal growth can be the disintegration and deterioration of fungal cytoplasmic parts and the degradation and dissolution of cytoplasm due to application of AGE as previously reported (Aala et al., 2014). Allicin has fungistatic and fungicidal effect and destroys the physical structure of fungal hyphal cell wall (Khan and Zhihui, 2010). Being physiologically active in microbial, plant and mammalian cells, allicin can inhibit the proliferation of fungi or kill cells outright in a dose dependent manner (Borlinghaus et al., 2014). The capability of physical damage to the mycelium and hyphal cell wall as well as the physiological activity inside the target species therefore support our findings that AGE possess inhibitive capacity against fungal growth. Another hypothesized mechanism to explain and support current findings might be the readily diffusion of allicin across a variety of phospholipid membranes (Miron et al., 2000) and due to its allylthio moiety, allicin preferentially reacts with free cysteine groups inside the fungal cells. Findings of Wallock-Richards et al. (2014) further support our postulations who reported that allicin containing AGE modified an essential *B. cenocepacia* catalytic cysteine residue and suggested a role for allicin as a general electrophilic reagent that targets protein. For a successful fungal infection, penetration into the plant cell and utilization of the nutrients found therein are the fundamental proceedings. Most of fungi use various cell wall degrading enzymes to penetrate into the host cellular compartments (Tonukari, 2003) and if these enzymes are restrained or disrupted, the ability to penetrate into and consequently colonize inside host body is impaired. Muhsin et al. (2001) reported that garlic bulb extracts significantly inhibited production of cell wall degrading enzymes in various fungi. A variety of organosulfur compounds such as DADS, DATS, Ajoenes etc., may contribute to the biological activity of garlic derived compounds (Avato et al., 2000; Rahman and Lowe, 2006; Yun et al., 2014). However, during decomposition of allicin into other compounds for example DADS, the antimicrobial property decrease (Borlinghaus et al., 2014). Therefore, the antifungal potency of the freshly prepared AGE employed in the current research can be attributed primarily to the contribution of its allicin content. Furthermore, in leaf disk bioassay, the disease severity % and severity index % was significantly reduced when the leaf disks were applied with AGE or pure allicin (AAS) and the effect was linear to the concentrations applied (**Figure 7**). Compared to the control, the highest concentration of both the AGE and AAS had the lowest disease severity % and severity index %. These findings also evidence the significant hindering force of allicin against fungal infection on the plant surface. A plausible explanation to understand the action of AGE against these fungi could be the physical demolition of fungal hyphae and the impaired secretion capability of cell wall degrading enzymes by these subject fungal strains may restrain the fungal infections. However, the exact chemistry involved, still remain enigmatic and further detailed approach such as proteomics or metabolomics to understand the bioactivity of AGE inside fungal biology as well as the interaction protocol of AGE with plant surface biology may unravel this mystery. Although AGE successfully inhibited fungal growth, we found differences among the colonial growth of the tested fungi, indicating the specific biological and genetic makeup of different genus that might show variance between growth patterns when subjected to the AGE concentrations.

HPLC analysis revealed considerable differences in the distribution of allicin content abundance. Allicin (Diallyl Thiosulfinate) is formed by the action of alliin-lyase (E.C.4.4.1.4) on alliin (*S*-allyl-L-cysteine sulphoxide; Curtis et al., 2004). Allicin content therefore reflects the alliin content of garlic bulb and can be used as a significant phytochemical trait for understanding the genetic diversity among various garlic ecotypes of different geographical origins. Studies on the phytochemical constituents of garlic lines have reported significant differences in the antioxidant activities and phytochemicals (Bhandari et al., 2014) which are in line with present findings. Although sexual propagation of garlic is possible, nearly all garlic in cultivation is propagated asexually by planting individual cloves. Thus, garlic frequently displays a high degree of genotypic plasticity that is likely to be dependent on the soil type, moisture, latitude, altitude, and agricultural practices (Horníčková et al., 2010). Ghani (2010) reported variable alliin and allicin content in garlic cultivars from Iraqi and French origins, suggesting genetic variation between the cultivars. Furthermore, our findings strongly corroborate previous reports that the total phenolic contents and antioxidant capacity of cultivar G074 and a few others are much stronger as compared to G105 (Chen et al., 2013). Current findings revealed that cultivar G025 bears the highest allicin content of 3.98 mg g^-1^, and can be justified from the study reported by Yun et al. (2014), who reported that range of allicin is different based on various geographical origin. Previous literature have shown difference in the allelopathic potential of various garlic cultivars (Xiao et al., 2012) and therefore support our hypothesis that the variance in allicin content could be attributed to the genetic diversity among different garlic cultivars. Moreover, current findings provide basis for genetic variation and breeding prospects for development of garlic lines capable of stronger pharmacological aspects. Few reports also confirms our findings that difference in allicin content provides basis for genetic diversity in allium species (Horníčková et al., 2010; Khar et al., 2011; Wang et al., 2014). Nonetheless, many commercial preparations are often standardized on the basis of sulfur-containing compounds, particularly to the Alliin or on the allicin yield (Barnes and Anderson, 2007), therefore current findings also lay foundation for isolation, identification and preparations of garlic from various Chinese cultivars on commercial basis.

In cucumber bioassay, the growth response of cucumber seedlings to the variable concentrations of AGE can be interpreted as the synergy and antagonism of AGE onto the growth of the subject plants. In order to understand the biological triggering of AGE, we selected morphological characters such as plant height, root length and stem diameter. Also, physiological parameters comprehensively related to stress conditions such as antioxidant enzymes and the MDA content were evaluated. It was observed that AGE at low concentrations, was conducive to cucumber seedlings height and root length whereas at higher concentration, their influence was suppressive. As previously reported, AGE’s foliar applications accelerate growth of receiver plants through stimulation of photosynthetic pigments and soluble sugar contents (Muhammad and Akladious, 2014). A stimulated photosynthesis system reflects an enhanced food factory which may have influenced cucumber height and root length consequently. During the last decade, the allelopathic potential of garlic has been documented to promote seed germination and seedling growth at low concentrations whereas, the effect is vice versa at higher concentrations (Yan-li et al., 2007; Xiao et al., 2012) and current findings are therefore in line with these reports. Garlic contain enzymes, vitamin B, vitamin C proteins, minerals such as Na, K, Zn, P, Mn, Mg, Ca, and Fe, carbohydrates, saponins, alkaloids, flavonoids, free sugars such as sucrose, fructose, and glucose (Otunola et al., 2010; Bhandari et al., 2014; El–Hamied and El-Amary, 2015) which offers a balanced proportion of nutritional dose for cucumber growth. Moreover, our findings revealed significant alterations in the antioxidant enzymes activities and the MDA content of cucumber seedlings when AGE was applied which indicate the conceivable bio-elicitation capability of garlic bulb extracts in the context of priming the antioxidant enzymes. We hereby advance our argument in two dimensions; activation of antioxidants and possible reactive oxygen species (ROS) regulatory mechanism in cucumber leading to enhanced growth conditions whereas and, the growth inhibition of cucumber seedlings at high concentrations of AGE. During plant growth, a sophisticated regulation mechanics of antioxidant enzymes and ROS is in continuum and many reports evidence the crosstalk of these two fundamentals to be vital for the biology of plants (Ahmad et al., 2008; Liu N. et al., 2014). When cucumber seedlings were applied with 150 μg mL^-1^ AGE, a significant increase in the abundance of SOD and CAT was observed compared with the control. SOD catalyzes the dismutation of O^2-^ into H_2_O_2_ and O_2_ while CAT and POD further transform H_2_O_2_ into H_2_O and O_2_. Thus, SOD and CAT serve in tandem as front-line antioxidant defenses in the plants (Racchi, 2013). Therefore, the observed alterations of these antioxidants resulted from various concentrations of AGE can be understood as possible ROS activation in cucumber seedlings. ROS are the byproducts of biological redox reactions (Arora et al., 2002). There is plethora of research debating ROS as stress indicator (Arora et al., 2002; Ahmad et al., 2008; Das and Roychoudhury, 2014). It’s vital activity in cellular development, however, is documented as well (Dunand et al., 2006; Liu N. et al., 2014). ROS are believed to be phytotoxic, but they are also known to play an important role in various key functions of the plants, particularly in promoting polysaccharide metabolism as well as cell wall loosening and elongation (Liu N. et al., 2014). In current findings, the altered levels of SOD and CAT infer the onset of stress like conditions in cucumber seedlings, indicating enhanced ROS levels. However, it seems to be equilibrium between the ROS and antioxidant system which possibly might prevent membrane lipid peroxidation, enabling cellular homeostasis and alternatively increase the growth of cucumber seedlings. Garlic as antioxidant has potential to modulate the ROS (Banerjee et al., 2003). Findings of Tian et al. (2012) support our hypothesis reporting increased antioxidant enzymes activity against ROS in marigold leaves during drought stress conditions. ROS distribution, however, also influence root development as reported earlier in *Arabidopsis* (Dunand et al., 2006) and therefore, the promoted root length observed in current findings could be due to the excited ROS that promoted the root development, while the excessive ROS would have been neutralized by the active antioxidant system. A progressive root development can offer enhanced channel of mineral uptake from the rhizosphere resulting in improved plant growth. Production of H_2_O_2_ in maize seedling as a defense strategy actively targeting the hyphae of *Colletotrichum graminicola* (Vargas et al., 2012), explain the idea of induced plant defense and offers support to our assumption that AGE application induces antioxidants is a trigger to ROS excitation in cucumber seedlings. Utilization of this excited state as induced resistance, however, requires further study. Garlic extracts were reported to induce priming of systemic resistance in cucumber seedlings against anthracnose (Inagaki et al., 2011) and our findings, therefore, are in agreement to this study suggesting that AGE induce the defense system of cucumber seedlings. AGE at the highest concentration (300 μg mL^-1^) however, resulted in stunted growth and decreased level of antioxidants whereas increased the abundance of MDA content in cucumber seedlings. Higher concentrations of garlic derived compounds have been reported to negatively affect the receiver crop growth (Zhi-hui, 2011; Han et al., 2013). It is possible that 300 μg mL^-1^ of AGE imposed oxidative stress on cucumber seedlings shifting the balance between prooxidative and antioxidative reactions in the favor of the former (Bartosz, 1997). The activity of antioxidant enzymes decreased whereas MDA content increased drastically at this stage. A plausible explanation could be that the overproduction of ROS cause oxidative burst, resulting peroxidation of membrane lipids and alternatively increasing the MDA content (Savicka and Škute, 2010). The increase in MDA content is a reflection of severe stress and our findings are in close agreement with (Hassan et al., 2015) who reported an increased level of MDA content in tomato leaves upon imposition of salt and water stress conditions. Increased MDA content was also reported as a stress condition observed in eggplants during successive cropping conditions (Wang et al., 2015). Therefore, it can be understood that AGE at 150 μg mL^-1^ regulate the prooxidative and antioxidative reactions in cucumber seedlings which is beneficial for enhanced growth, whereas the overdose of AGE (300 μg mL^-1^) cause lipid peroxidation and impose stress on the cucumber seedlings. However, more appropriate and targeted approaches are needed to understand the actual biostimulation of AGE as induced defense chemical and identify its molecular pattern inside plant biology.

## Conclusion

Our findings strongly confirm the antifungal potential of garlic extracts and provide basis for preparations of potent bio fungicide with broad spectrum potential. Current findings suggest that Phytoalexin allicin is the primary among various antifungal constituent of AGE and the abundance is diversified among different cultivars. Furthermore, this antifungal potency is not solely attributed to the allicin content since many other organosulfur compounds maybe be involved alongside. Therefore, in the future, more careful observations will be carried out to isolate individual allelochemicals of garlic bulb extract and identify their particular bioactivity. The diversity in allicin abundance between various cultivars offer a significant phytochemical trait to explore genetic diversity in garlic. Moreover, current research findings lay foundation for conservation of garlic cultivars bearing strong allicin content for breeding purposes and pharmaceutical applications. Leaf disk bioassay allow us to consider elaborated evaluation of AGE as botanical fungicide in specialized horticultural situations where fungal infections hamper the production. Future studies will be carried out to understand the mechanism of antifungal activity of AGE on the plant surface employing advanced microscopic and physiological assessments of the plant as well as fungal biology. Furthermore, research need to be done employing advanced proteomics and metabolomics to clarify the interaction of AGE with fungal biology and understand the antifungal process accordingly. Bioassay on cucumber plants show that garlic bulb extracts are physiologically active inside the receiver plants altering the antioxidant mechanisms which result in advanced growth. Current results hence provide a platform to explore the mechanism involved on molecular levels. The antioxidant activity of cucumber could be interpreted as advanced or induced resistance and further research will be carried out to explore the possibility of garlic bulb extracts as bio-stimulator for induced resistance against fungal pathogenicity. It is quite possible that more than one constituent may be involved in the biostimulation of antioxidant system in the receiver plants, therefore in future, more elaborate and specified approaches need to be carried out to understand bioactive components of AGE and identify their particular mechanism of action in plant defense activation.

## Author Contributions

All authors made contribution to the experiment work and manuscript write up. SH made substantial contribution to the design of the work, acquisition, analysis, and interpretation of data and drafting the manuscript. ZC made contribution to the conception and design of the work and critically revision of the article for important intellectual content. HA and MA made contribution to the acquisition of the data. MW and XC made contribution to the design of the work. All authors made contribution to the approval of the final version of the manuscript to be published and agreed to be accountable for all aspects of the work in ensuring that questions related to the accuracy or integrity of any part of the work are appropriately investigated and resolved.

## Conflict of Interest Statement

The authors declare that the research was conducted in the absence of any commercial or financial relationships that could be construed as a potential conflict of interest. The reviewer VG and handling Editor declared their shared affiliation, and the handling Editor states that the process nevertheless met the standards of a fair and objective review.
